# Validation of a Case-Finding Algorithm for Identifying Patients with Non-small Cell Lung Cancer (NSCLC) in Administrative Claims Databases

**DOI:** 10.3389/fphar.2017.00883

**Published:** 2017-11-30

**Authors:** Ralph M. Turner, Yen-Wen Chen, Ancilla W. Fernandes

**Affiliations:** ^1^HealthCore, Inc., Wilmington, DE, United States; ^2^Janssen Pharmaceuticals, Inc., Titusville, NJ, United States; ^3^AstraZeneca Pharmaceuticals LP, Gaithersburg, MD, United States

**Keywords:** algorithm, claims data, non-small cell lung cancer, sensitivity, small cell lung cancer, specificity

## Abstract

**Objective:** To assess the validity of a treatments- and tests-based Case-Finding Algorithm for identifying patients with non-small cell lung cancer (NSCLC) from claims databases.

**Data sources:** Primary data from the HealthCore Integrated Research Environment (HIRE)-Oncology database and the HealthCore Integrated Research Database (HIRD) were collected between June 1, 2014, and October 31, 2015.

**Study design:** A comparative statistical evaluation using receiver operating characteristic (ROC) curve analysis and other validity measures was used to validate the NSCLC Case-Finding Algorithm vs. a control algorithm.

**Data collection:** Patients with lung cancer were identified based on diagnosis and pathology classifications as NSCLC or small-cell lung cancer. Records from identified patients were linked to claims data from Anthem health plans. Three-month pre-index and post-index data were included.

**Principal findings:** The NSCLC Case-Finding Algorithm had an area under the curve (AUC) of 0.88 compared with 0.53 in the control (*p* < 0.0001). Promising diagnostic accuracy was observed for the NSCLC Case-Finding Algorithm based on sensitivity (94.8%), specificity (81.1%), positive predictive value (PPV) (95.3%), negative predictive value (NPV) (79.6%), accuracy (92.1%), and diagnostic odds ratio (DOR) (78.8).

**Conclusions:** The NSCLC Case-Finding Algorithm demonstrated strong validity for distinguishing patients with NSCLC from those with SCLC in claims data records and can be used for research into NSCLC populations.

## Introduction

Lung cancer is the leading cause of cancer-related deaths in both men and women, and is a heterogeneous malignancy composed of several subtypes (Siegel et al., 2017). Approximately 80–85% of lung cancers are classified as non-small cell lung cancer (NSCLC) and the remaining 15–20% as small cell lung cancer (SCLC) (American Cancer Society, 2016). These two subtypes of lung cancer have distinct genetic alterations and prognoses, requiring different treatment modalities to be used for NSCLC vs. SCLC (NCCN NSCLC, 2017a; NCCN SCLC, 2017b). It is, therefore, important to be able to distinguish between these subtypes of lung cancer when investigating therapy options, clinical outcomes, and associated costs.

Secondary data sources, such as administrative claims data, cancer registries, and electronic medical records, provide valuable information to complement results from randomized clinical trials that can be used to profile care patterns, measure patient outcomes, and estimate cancer-related costs (Schulman et al., 2013). However, for these databases to be considered as a reliable source of information for research studies, it is important that patients with the subtype of cancer or disease of interest can be identified correctly.

Coding systems such as the International Classification of Diseases, Ninth Revision, Clinical Modification (ICD-9-CM) or the ICD-10-CM are typically used to identify patients with a specific disease or condition in secondary data analyses. In some cases, supplemental laboratory, histological, or biomarker data may be used to diagnose specific cancers, but the selection process for correctly identifying the population of interest has to be validated. If the process is not accurate, the patient cohort selected may not be reflective of the larger population of interest (poor sensitivity) or may contain large numbers of patients who do not have the disease (poor specificity) (Schulman et al., 2013), making the results of such secondary analyses questionable.

Neither the ICD-9-CM nor the ICD-10-CM coding system differentiates between SCLC and NSCLC, which creates a significant challenge for researchers using large claims databases to study these two subtypes of lung cancer. For such research, using an existing algorithm or creating a new algorithm based on pertinent diagnostic, procedure, and drug codes might serve to accurately distinguish between NSCLC and SCLC populations.

Duh et al. developed an algorithm to identify cases of SCLC from among lung cancer cases in administrative claims databases, based on the American Cancer Society (ACS) and the National Comprehensive Cancer Network (NCCN) treatment guidelines, as well as clinical experience from a retrospective claims database analysis (Duh et al., 2008). The original algorithm was designed to identify patients with SCLC; however, recently, a few studies have modified the algorithm by reversing the inclusion and exclusion criteria to identify patients with NSCLC (Karve et al., 2014; Fernandes et al., 2016a). In this modified algorithm (Modified Duh Algorithm), the inclusion criteria contain procedures and chemotherapies used for patients with NSCLC, and the exclusion criteria consist of chemotherapy regimens applied to patients with SCLC (Turner et al., 2015, in press; Fernandes et al., 2016a,b; Karve et al., 2016). Although often used, the Modified Duh Algorithm has not been formally validated for accuracy for NSCLC populations. Based on these facts, we have developed a new algorithm to identify NSCLC cases from heterogeneous lung cancer populations—the NSCLC Case-Finding Algorithm. Development of the NSCLC Case-Finding Algorithm began with the Modified Duh Algorithm, and updating the treatments and tests that make up the algorithm based on updated cancer treatment guidelines. In addition, the scoring system of the NSCLC Case-Finding Algorithm was organized to reflect the goal of identifying patients with NSCLC from heterogeneous lung cancer populations. The objective of the current study was to assess the validity of the NSCLC Case-Finding Algorithm using data from a clinical database.

## Methods

### Data source

This study used lung cancer cases identified from the HealthCore Integrated Research Environment (HIRE)-Oncology clinical database that were linked with the HealthCore Integrated Research Database (HIRD).

The HIRE-Oncology clinical database is a product of the Clinical Cancer Quality Program for Anthem (AIM Specialty Health®, Deerfield, IL), which compares planned cancer treatment regimens against evidence-based clinical criteria such as efficacy, toxicity profile, and cost (Malin et al., 2015). Clinical information collected for the program is integrated with the medical and pharmacy claims data contained within the HIRD and, for patients with cancer, including lung malignancies, includes: cancer type (ICD-9 or ICD-10 and description); cancer stage; tumor biomarkers; line of treatment (e.g., adjuvant/postoperative; first-line, second-line, third-line, later-line; maintenance); height and weight; treatment regimen details with individual drugs and doses; and Eastern Cooperative Oncology Group (ECOG) performance status.

The HIRD contains longitudinal medical and pharmacy claims data on ~43 million members of Anthem health plans from across the United States in regions defined as Northeast, Midwest, South, and West. Member enrollment, medical care (professional and facility claims), outpatient prescription drug use, outpatient laboratory test results data, and healthcare utilization may be tracked for health plan members in the HIRD dating back to January 2006. The database includes additional claims information from a commercially insured United States population obtaining healthcare under schemes such as health maintenance organization plans, point of service plans, preferred provider organizations, indemnity plans, and Medicare supplemental plans.

### Study design

The NSCLC Case-Finding Algorithm was compared with a control algorithm (defined later in the text) using information collected from the HIRD and the HIRE-Oncology database to determine the properties of the algorithm compared with one based on all available lung cancer treatments and diagnostic tests. The molecular pathology information from the HIRE-Oncology database that specified NSCLC vs. SCLC status for each patient served as the validation criterion.

This study complied with all state and federal laws and regulations related to the privacy and security of individually identifiable health information, including the Health Insurance Portability and Accountability Act. Patient identity was masked throughout using a limited data set format. Under the terms of the research exception provisions of the Privacy Rule, 45 CFR 164.514(e), institutional review board approval was not a requirement in this study.

### Patient selection

All adult patients (aged ≥18 years) within the HIRD and participating in the Anthem Cancer Care Quality Program diagnosed with lung cancer during the intake period (June 1, 2014, to October 31, 2015) and who had received ≥1 chemotherapy/radiation or lung cancer surgery regimens were eligible for this study. Patient index date was defined as the earliest date of precertification for lung cancer in the HIRE-Oncology database during the intake period. Patients were required to be continuously enrolled within the health plan for ≥3 months before and after the index date. Records from patients identified in the HIRE-Oncology database were linked to corresponding administrative claims information retrieved from the HIRD. Eligible patients with lung cancer were identified in the HIRE-Oncology database based on a diagnosis of lung cancer registered under the “Cancer Type” variable with a molecular classification of NSCLC or SCLC. Therefore, at minimum, the start of the observation period was March 1, 2014, and the end date was January 31, 2016. To ensure maximum data capture, data available beyond this duration were included when available between June 1, 2013, and April 30, 2016. Patients were excluded if information on their histology/pathology status specifying NSCLC vs. SCLC was missing in the HIRE Oncology database.

### NSCLC case-finding algorithm

Inclusion criteria for the existing Modified Duh Algorithm were based on the first-line chemotherapy regimens administered to patients with NSCLC, and exclusion criteria included procedures, surgeries, and chemotherapies administered to patients with SCLC as recommended by the 2006 NCCN Guidelines (American Cancer Society and NCCN, 2006). The NSCLC Case-Finding Algorithm described in the current article was developed based on the existing Modified Duh Algorithm and updated to include first-line treatments and test recommendations for patients with NSCLC and SCLC as specified by the 2015 ACS (American Cancer Society, 2015) and 2016 NCCN guidelines (Ettinger et al., 2016) as well as ICD-10 codes. The inclusion and exclusion criteria developed for the NSCLC Case-Finding Algorithm are presented in Table 1. The generic product identifier (GPI) codes, Healthcare Common Procedure Coding System (HCPCS) codes, ICD-9 and ICD-10 Procedure codes, and Current Procedural Terminology (CPT) codes used to identify each inclusion and exclusion criterion can be found in Appendix A in Supplementary Material.

**Table 1:** Inclusion and exclusion first-line treatment and tests criteria.

	**NSCLC Case-Finding Algorithm**	**Control algorithm**
**SMALL CELL LUNG CANCER EXCLUSION CRITERIA**		
Cisplatin and etoposide	x	x
Cisplatin and irinotecan	x	x
Carboplatin and etoposide	x	x
Topotecan	x	x
Cyclophosphamide, doxorubicin, and vincristine	x	x
Carboplatin and irinotecan	x	x
Temozolomide	x	x
Ifosfamide	x	x
Bendamustine	x	x
**NON-SMALL CELL LUNG CANCER INCLUSION CRITERIA**		
PET scan imaging	x	x
Lung removal or resection surgery	x	x
Carboplatin and paclitaxel	x	x
Carboplatin and gemcitabine	x	x
Carboplatin and vinorelbine	x	x
Cisplatin and gemcitabine	x	x
Cisplatin and vinorelbine	x	x
Gemcitabine and vinorelbine	x	x
Paclitaxel		x
Docetaxel		x
Erlotinib	x	x
Gemcitabine		x
Etoposide		x
Vinorelbine		x
Irinotecan		x
Cisplatin and docetaxel	x	x
Cisplatin and pemetrexed	x	x
Cisplatin and paclitaxel	x	x
Cisplatin and vinblastine	x	x
Carboplatin and pemetrexed	x	x
Carboplatin and docetaxel	x	x
Gemcitabine and docetaxel	x	x
Bevacizumab, carboplatin, and paclitaxel	x	x
Bevacizumab, carboplatin, and pemetrexed	x	x
Bevacizumab, cisplatin, and pemetrexed	x	x
Afatinib	x	x
Gefitinib	x	x
Osimertinib	x	x
Crizotinib	x	x
Alectinib	x	x
Ceritinib	x	x
Pemetrexed		x
Abraxane (nab-paclitaxel)	x	x

*PET, position emission tomography*.

### Control algorithm

To provide a baseline for comparison for the NSCLC Case-Finding Algorithm, the complete list of the 2015 ACS (American Cancer Society, 2015) and 2016 NCCN (Ettinger et al., 2016) recommended treatments for NSCLC and SCLC were combined and used as a control algorithm for this study (Table 1). For this control algorithm, all tests and first-line treatments (i.e., also those for SCLC) were used as inclusion criteria for NSCLC, and no exclusion criteria were specified. We hypothesized that the control algorithm would not be able to distinguish between lung cancer populations, but would provide a comparative baseline to assess the validity of the NSCLC Case-Finding Algorithm.

### Study measures

#### Descriptive variables: definition and assessment

To characterize the patient population, demographic and comorbid illness variables were summarized from the HIRD, and clinical variables were summarized from the HIRE-Oncology database.

#### Analysis plan

The analysis plan follows the guidelines and methods for the statistical classification of dichotomously scored medical tests recommended by Pepe (2003). Diagnostic accuracy of the algorithms was assessed using the following statistical measures: sensitivity, specificity, positive predictive value (PPV), negative predictive value (NPV), accuracy, diagnostic odds ratio (DOR), and area under the curve (AUC).

Non-parametric receiver operating characteristic (ROC) curve analysis was used to select the best-performing algorithm. The Stata Receiver Operating Characteristic Curve Comparison (ROCCOMP) analysis for comparing ROC curves from the same sample of patients was used. ROCCOMP uses the DeLong et al. (1988) and Hanley and McNeil (1983) approaches for comparing ROC curves based on correlated data. It provides an omnibus test of the equality of the AUC of the algorithms being compared and reports all summary statistics shown in the current article. In addition, the Stata logistic regression procedure was used to test the sensitivity and robustness of the NSCLC Case-Finding Algorithm across the factors of age, gender, cancer stage, body mass index (BMI), Deyo–Charlson Comorbidity Index (DCI) score, and commercial vs. Medicare supplemental insurance. A hierarchical approach for hypothesis testing was used. The covariate factors were entered in the first step of the analysis in order to allow them to account for maximum variance in the validation criterion. The NSCLC Case Finding Algorithm was entered in the second step, and its incremental contribution to the overall variance of the histology/pathology status was assessed. Follow-up logistic regression analyses were conducted to assess interactions between the covariates and the NSCLC Case Finding Algorithm.

## Results

### Patient characteristics

The overall sample population consisted of 1,353 patients with lung cancer, among whom 270 (20.0%) were classified as SCLC and 1,083 (80.0%) as NSCLC according to the pathology information in the HIRE-Oncology database (Table 2). Mean age of the SCLC cohort was 60.5 years and of the NSCLC cohort was 59.6 years, and the median age was 60 years for both cohorts. The SCLC cohort was 50.7% male and 49.3% female, whereas the NSCLC cohort was 49.8% male and 50.2% female. Average DCI scores were 7.6 and 7.4 for the SCLC and NSCLC cohorts, respectively. Mean BMI was indicative of patients being slightly overweight in both cohorts, with the SCLC average at 26.9 kg/m^2^ and the NSCLC average at 26.0 kg/m^2^. The majority of patients with SCLC (84.4%) or NSCLC (90.1%) had Stage IIIA through IV disease. The patient population resided in all regions of the United States, although fewer patients came from the Northeast than from other regions. Only 3% of patients were enrolled in Medicare supplemental plans, suggesting that most patients had commercial insurance.

**Table 2:** Patient demographic and cancer features at index date.

	**Total *N* = 1353**	
	**SCLC**	**NSCLC**
Number of patients, *n* (%)	270 (20.0)	1083 (80.0)
Age, years, mean ± *SD* (median)	60.5 ± 7.1 (60.0)	59.6 ± 8.7 (60.0)
Gender, *n* (%)		
Male	137 (50.7)	539 (49.8)
Female	133 (49.3)	544 (50.2)
Medicare supplemental, *n* (%)	8.0 (3.0)	33.0 (3.0)
Region of residence, *n* (%)		
Northeast	38 (14.1)	186 (17.2)
Midwest	90 (33.3)	330 (30.5)
South	84 (31.1)	295 (27.2)
West	58 (21.5)	272 (25.1)
Deyo–Charlson Comorbidity Index, mean ± *SD* (median)	7.6 ± 2.9 (8.0)	7.4 ± 2.8 (8.0)
BMI, mean ± *SD* (median)	26.9 ± 6.7 (26.2)	26.0 ± 6.0 (25.5)
Cancer stage, *n* (%)		
0	2 (0.7)	0 (0)
IA	5 (1.9)	3 (0.3)
IB	1 (0.4)	12 (1.1)
IIA	3 (1.1)	53 (4.9)
IIB	0 (0)	36 (3.3)
IIIA	10 (3.7)	136 (12.6)
IIIB	13 (4.8)	84 (7.8)
IV	205 (75.9)	755 (69.7)
Limited	31 (11.5)	4 (0.4)

*Baseline period: index date −90 to index date −1*.

*BMI, body mass index; NSCLC, non-small cell lung cancer; SCLC, small cell lung cancer; SD, standard deviation*.

### Accuracy analyses

Table 3 presents the two-way cross-tabulations of the control algorithm (top of table) and the NSCLC Case-Finding Algorithm (bottom of table) with the HIRE-Oncology validation criterion. Sensitivity of the NSCLC Case-Finding Algorithm was 94.8%, specificity was 81.1%, PPV was 95.3%, NPV was 79.6%, overall accuracy was 92.1%, and the DOR was 78.8. The control algorithm's sensitivity was 7.4%, specificity was 14.4%, PPV was 25.7%, NPV was 3.7%, overall accuracy was 8.8%, and the DOR was 0.01. Therefore, the control algorithm provided no improvement in knowledge of NSCLC status and actually performed worse than categorization by chance alone; whereas, the quality of the diagnostic accuracy of the NSCLC Case-Finding Algorithm appears strong.

**Table 3:** Algorithm classification^a^.

			**HIRE-oncology criterion**		**Total**
			**SCLC**	**NSCLC**	
**CONTROL ALGORITHM**					
Control algorithm	SCLC	*n* (%)	39 (2.9)	1003 (74.1)	1042 (77.0)
	NSCLC	*n* (%)	231 (17.1)	80 (5.9)	311 (23.0)
Total		*n* (%)	270 (20.0)	1083 (80.0)	1353 (100.0)
**NSCLC CASE-FINDING ALGORITHM**					
NSCLC Case-Finding Algorithm	SCLC	*n* (%)	219 (16.2)	56 (4.1)	275 (20.3)
	NSCLC	*n* (%)	51 (3.8)	1027 (75.9)	1078 (79.7)
Total		*n* (%)	270 (20.0)	1083 (80.0)	1353 (100.0)

a *Percents are percent of Total N (1353)*.

### Model comparisons

**Figure 1:**
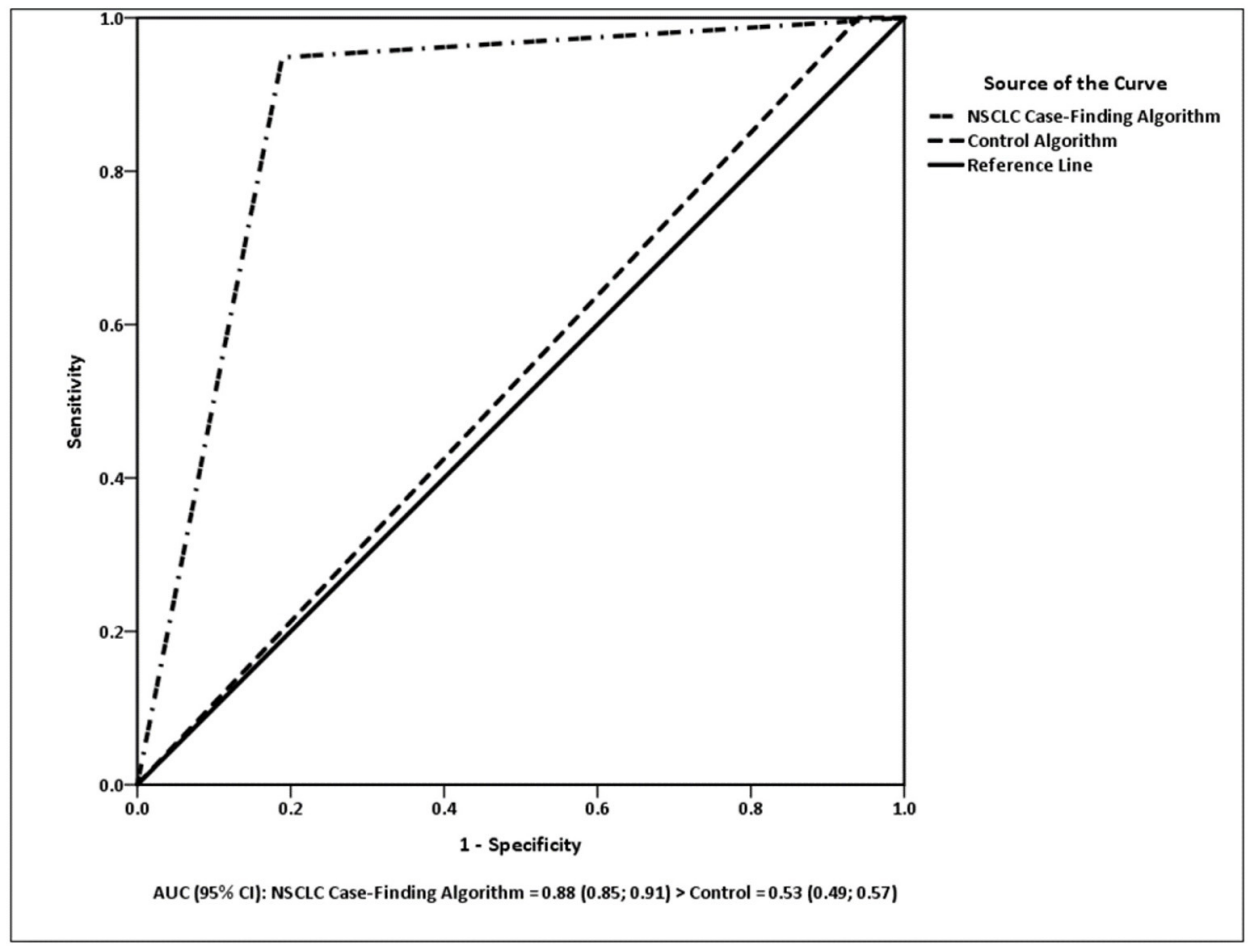
AUC (95% CI): NSCLC Case-Finding Algorithm = 0.88 (0.85; 0.91) > control = 0.53 (0.49; 0.57).

The ROC curves for the algorithm models are presented in Figure 1. There was a significant difference between the algorithms in the ROC analysis [${\chi \;}_{\left(\text{df}\;=\;2\right)}^{2}$ = 663.48, *p* < 0.0001]. The NSCLC Case-Finding Algorithm had a higher AUC (0.88; 95% CI 0.85, 0.91) than the control (0.53; 95% CI 0.49, 0.57). The AUC for the control algorithm was not significantly different from 0.5 (*p* = 0.131), but the NSCLC Case-Finding Algorithm was significantly larger than 0.5 (*p* = 0.0001).

A hierarchical logistic regression analysis was used to assess the impact of the covariates on the functioning of the NSCLC Case Finding Algorithm. The first model assessed how well age, gender, cancer stage, BMI, DCI score, and commercial vs. Medicare supplemental insurance predicted the validation criterion of HIRE-Oncology histology/pathology status. The pseudo *R*^2^ was 0.007 (*p* = 0.164) for this model, and none of the covariates were significant predictors of the validation criterion: age (*p* = 0.175), gender (*p* = 0.832), cancer stage (*p* = 0.297), BMI (*p* = 0.072), DCI score (*p* = 0.260), and commercial vs. Medicare supplemental insurance (*p* = 0.788). In the next step, the NSCLC Case Finding Algorithm was added to the model to estimate the incremental improvement in the multiple pseudo *R*^2^. This logistic regression analysis obtained a pseudo *R*^2^ = 0.531 (*p* = 0.0001). The change in the pseudo *R*^2^ attributable to the NSCLC Algorithm, after controlling for the covariates, was 0.524 (0.531 minus 0.007; *p* = 0.0001). The NSCLC Algorithm was a significant predictor of the validation criterion (*p* = 0.0001), but, as before, none of the covariates was a statistically significant predictor: age (*p* = 0.618), gender (*p* = 0.734), cancer stage (*p* = 0.201), BMI (*p* = 0.092), DCI score (*p* = 0.697), and commercial vs. Medicare supplemental insurance (*p* = 0.273). The AUC for the NSCLC Case-Finding Algorithm remained unchanged 0.88 (95% CI 0.83, 0.91). We subsequently tested for interaction of the covariates with the NSCLC Case-Finding Algorithm. None of the interactions were statistically significant: age (*p* = 0.44), gender (*p* = 0.09), cancer stage (*p* = 0.33), BMI (*p* = 0.51), DCI score (*p* = 0.23), and commercial vs. Medicare supplemental insurance (*p* = 0.76). There was no evidence that the covariates affected the functioning of the NSCLC Case-Finding Algorithm.

## Discussion

In this analysis, an algorithm designed to identify NSCLC cases from among a pool of patients diagnosed with lung cancer was tested for accuracy, using the clinical/pathological data from the HIRE-Oncology database as the validation criterion. The accuracy of the NSCLC Case-Finding Algorithm was compared with that of a control algorithm to provide greater context for the findings. The results show that, first, the AUC was significantly stronger than the AUC of the control algorithm. Second, using the NSCLC Case-Finding Algorithm increased the odds to 78.8 for correctly identifying patients with NSCLC compared with the baseline odds of 4.0 when using the control algorithm. The NSCLC Case-Finding Algorithm's validity statistics were all strong. The primary validity results obtained for the NSCLC Case-Finding Algorithm were supported by the sensitivity analysis, where the AUC remained the same (0.880) when controlling for age, gender, cancer stage, BMI, DCI score, and commercial vs. Medicare supplemental insurance, and none of these variables interacted with the NSCLC Algorithm or accounted for significant variance in the validation criterion.

According to the national population reports based on the Surveillance, Epidemiology, and End Results (SEER) database, between 2010 and 2014 the majority of patients with NSCLC were aged 65 years or older at the time of diagnosis (Howlader et al., 2014). The median age of patients in this analysis was 60 years, which is slightly lower than that in SEER, potentially because of the high proportion of commercially insured (and thus younger) patients in the overall HIRD compared with in the SEER population. The percentage of females in this study sample (50.2%) was similar to that in the SEER population (47.3%) (Howlader et al., 2014) as well as that in the full HIRE-Oncology database (47.2%) (Barron et al., 2016), supporting the representativeness of the study population. However, neither age nor gender nor any additional variables tested within this sample affected the functioning of the NSCLC Case-Finding Algorithm and none of these variables was a statistically significant predictor.

Although the NSCLC Case-Finding Algorithm was superior to the control algorithm assessed in this study, there are limitations. First, the data were obtained from administrative claims, which may contain undetected coding errors. Second, because all patients included in the study were members of large US-based commercial health insurance plans, these results may not be generalizable to patients with other types of health insurance, possibly because of restrictions on certain types of therapies, or to patients treated outside the United States in regions that do not follow the same treatment guidelines. Third, the NSCLC Case-Finding Algorithm was based on tests and treatments received during the initial days following diagnosis. The algorithm did not consider second-, third-, or later-line treatments for determining classification. Although the NSCLC Case Finding Algorithm demonstrated excellent sensitivity (94.8%) and specificity (81.1%), it did classify a small proportion of patients with SCLC as NSCLC, which was likely due to patients with NSCLC and those with SCLC receiving many of the same treatments, making the task of discriminating between the two cancer subtypes difficult for treatment algorithms. As new therapies enter the treatment landscape for NSCLC, these may also be added to the algorithm to improve its accuracy. Finally, it should be noted that the NSCLC Case-Finding Algorithm for identifying patients with NSCLC should not be used in studies focused on characterizing treatment patterns, because the algorithm uses treatments to distinguish between patients with NSCLC and those with SCLC.

There is a need for continued research to improve the specificity of the NSCLC Case-Finding Algorithm and to test it over a broader range of patient populations. Accurately distinguishing between different lung cancer subtypes would make it possible to conduct claims-based oncology research specific to NSCLC and SCLC populations. Other studies have investigated the sensitivity of claims-based algorithms in the literature (McBean et al., 1994; McClish et al., 1997; Setoguchi et al., 2007). In the McBean et al., McClish et al., and Setoguchi et al. studies, the accuracy of administrative codes for lung cancer was compared with cancer registry records from patients eligible for Medicare, and the reported sensitivity of administrative codes ranged from 56 to 90% (McBean et al., 1994; McClish et al., 1997; Setoguchi et al., 2007). Ramsey et al. examined the sensitivity of administrative claims based on Medicaid, Medicare, and commercial health plans to identify NSCLC, and reported sensitivities of 51, 88, and 99%, respectively (Ramsey et al., 2009). Finally, Whyte et al. investigated the identification of patients with lung cancer from a healthcare claims database using ICD-9 codes in combination with tumor-specific algorithms, and reported sensitivity and specificity of 55 and 85%, respectively (Whyte et al., 2015). Together, these studies demonstrate that patient identification is affected by the database and algorithm used, and the sensitivity ranges observed with the NSCLC Case-Finding Algorithm are consistent with previously published studies.

At minimum, future studies on NSCLC that employ the NSCLC Case-Finding Algorithm, or any other algorithm, should assess and report the ROC and validity statistics associated with their application to allow readers to evaluate the results based on a good understanding of the patient composition included in the analysis.

There have been improvements in obtaining access to clinical data in oncology, such as with the HIRE-Oncology and the SEER-Medicare databases. However, while these databases are an option for specific populations, other databases may not have clinical data associated with claims readily available to use for understanding treatment outcomes and potentially influence treatment decisions/policies. In the absence of obtaining clinical data, the NSCLC Case-Finding Algorithm offers a reliable and valid technique to identify patients with NSCLC from within large databases of patients with lung cancer.

In summary, results from this study demonstrate that the NSCLC Case-Finding Algorithm may be useful for identifying patients with NSCLC from large US commercial claims-based databases for research purposes. Compared with the control algorithm and potential confounders, such as age or stage of cancer, the NSCLC Case-Finding Algorithm demonstrated strong sensitivity, specificity, PPV, NPV, accuracy, and a higher AUC.

## Author contributions

RT led the conception of the work, design of the work, analysis of data, interpretation of data, creation of tables and figures, and drafting and critical revision of the work. Y-WC collaborated on the conception of the work, design of the work, analysis of data, interpretation of data, creation of tables, and critical revision of the work. AF collaborated on the conception of the work, design of the work, and drafting and critical revision of the work.

### Conflict of interest statement

RT: Research support from AstraZeneca; Employment at HealthCore, Inc. Y-WC: Research support from AstraZeneca; Employment at HealthCore, Inc. AF: Stockholder and employment at AstraZeneca.
