# Supplementary material for: Validation of a Case-Finding Algorithm for Identifying Patients with Non-small Cell Lung Cancer (NSCLC) in Administrative Claims Databases
Source: Front Pharmacol. 2017 Nov 30;8:883. doi: 10.3389/fphar.2017.00883 (PMC5714924; doi:10.3389/fphar.2017.00883)
Supplement: Supplementary file 1 [file AppendixA.pdf]

APPENDIX A. Codes for NSCLC Case-Finding Algorithm Scoring (Schulman et al., 2013)

Therapy/Scan	GPI Code	HCPCS Code	ICD-9 Procedure Code	ICD-10 Procedure Code	CPT Code
PET scan		G0210-G0211, G0126, G0212, G0234			
Lung removal or resection surgery			32.0x, 32.1, 32.9x	0B5x, 0BBx, 0BTx	32440-32525
Abraxane (paclitaxel protein bound)	21500012201920	J9264			
Afatinib	21534006x				
Alectinib	21534007100120				
Bendamustine	2110000910x	J9033			
Bevacizumab	21335020x	J9035, Q2024			
Carboplatin	21100015x	J9045			
Ceritinib	21534014000130				
Cisplatin	21100020x	J9060			
Crizotinib	215340150001x				
Cyclophosphamide	21101020x	J9070, J8530			
Docetaxel	21500005x	J9171			
Doxorubicin	21200040x	Q2049, Q2050, J9000			
Erlotinib	21534025x				
Etoposide	21500010x	J8560, J9181, J9182, C9414, C9425			
Gefitinib	21534030x	J8565			
Gemcitabine	21300034x	J9201			
Ifosfamide	21101025x	J9208			
Irinotecan	21335010x	J9206			
Osimertinib	215340652003x				
Paclitaxel	215000120013x	J9267			
Pemetrexed	21300053x	J9305			
Temozolomide	21104070x	J8700, J9328			
Topotecan	21550080x	J9351, J8705			
Vinblastine	21500030x	J9360			

Vincristine	21500020x	J9370, J9371			
Vinorelbine	21500050x	J9390			

CPT, Current Procedural Terminology; GPI, generic product identifier; HCPCS, Healthcare Common Procedure Coding System; PET, position emission tomography.
